# *Philometroides giginosantoroi* n. sp. (Nematoda: Philometridae), a new muscle-infecting species in the Mediterranean moray (*Muraena helena*) revealed using integrative taxonomy

**DOI:** 10.1017/S0031182024000581

**Published:** 2024-05

**Authors:** Alejandro López-Verdejo, Flavia Occhibove, Barbara degli Uberti, Fabio Crocetta, Mario Santoro

**Affiliations:** 1 Department of Integrative Marine Ecology, Stazione Zoologica Anton Dohrn, Villa Comunale 1, 80121 Naples, Italy; 2 Marine Zoology Unit, Cavanilles Institute of Biodiversity and Evolutionary Biology, University of Valencia, C/Catedrático José Beltrán 2, 46980 Paterna, Spain; 3 Istituto Zooprofilattico Sperimentale del Mezzogiorno, Via Salute 2, 80055 Portici, Italy; 4 NBFC, National Biodiversity Future Center, Piazza Marina 61, 90133 Palermo, Italy

**Keywords:** dracunculoids, fish parasites, integrative taxonomy, nematodes, Tyrrhenian Sea

## Abstract

*Philometroides* Yamaguti, 1935 is a genus of dracunculoid nematodes of the family Philometridae Baylis & Daubney, 1926 comprising tissue-infecting species worldwide. In the present study, a new species of *Philometroides* is described from the Tyrrhenian Sea (central-western Mediterranean Sea) using integrative approaches such as light and scanning microscopy, histopathology and 18S rRNA sequencing. *Philometroides giginosantoroi* n. sp. found in the skeletal muscles of the Mediterranean moray *Muraena helena* can be distinguished from its congeners by a combination of morphological traits and in particular by having the oral aperture with 3 large sclerotized triangular oesophageal teeth. The molecular analyses and phylogenetic reconstructions support its status as a new taxon and placed it within a clade of tissue-infecting species, although also confirmed mismatches in the generic assignment of several species. *Philometroides giginosantoroi* n. sp. is the second species of the genus found in the Mediterranean Sea and in general in the European marine waters and the third species of the family known to infect the family Muraenidae.

## Graphical abstract

**Figure:**
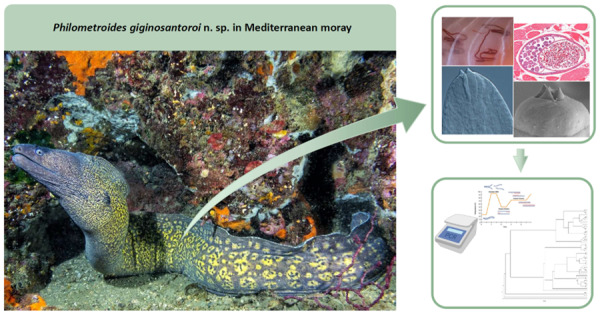

## Introduction

Philometridae Baylis & Daubney, 1926 is a family of dracunculoid nematodes mostly comprising gonad, body cavity and tissue-infecting species restricted to teleost fishes. Members of the family are characterized by a simple mouth surrounded by a variable number of cephalic papillae arranged in 2 circles; the buccal capsule is generally reduced or absent, and have an oesophagus, often bulbously inflated at its anterior end, provided with a large oesophageal gland (Moravec, 2004, 2006, 2024). Their life cycles involve copepods as intermediate hosts and fishes as paratenic and definitive hosts (Moravec, 2004). Male philometrids are considerably smaller and have a shorter life span than females (Moravec, 2004, 2006, 2024). A recent experimental study revealed that, in their definitive host, females of *Philometroides seriolae* (Ishii, 1931) Yamaguti, 1935 can take more than a year to be able to produce and disseminate larvae (Ogawa *et al*., 2023).

With a total of 217 valid species, the genus-level classification within Philometridae remains among the most problematic and unsatisfactory in the phylum Nematoda (Moravec and de Buron, 2013; Moravec, 2024). Their taxonomic diagnoses mostly rely on female morphology, as males are unknown for most species and even genera, or on molecular tools (Moravec, 2004, 2006, 2024).

The Mediterranean moray *Muraena helena* Linnaeus, 1758 (Anguilliformes: Muraenidae) is a nocturnal and territorial carnivorous species distributed throughout the whole Mediterranean Sea and the eastern Atlantic and mainly feeding on fish, crustaceans and cephalopods (Matić-Skoko *et al*., 2014; Sallami *et al*., 2014). Its meat is used and appreciated for human consumption since the Roman Empire, when it was farmed and raised in specific fishponds especially in the Gulf of Naples (Italy, Tyrrhenian Sea) (Pesando and Stefanile, 2016). Notwithstanding that, data on its helminth fauna are scarce and mostly limited to descriptions or reports of few specialist gastrointestinal trematodes (Bartoli *et al*., 2003, 2005; Bartoli and Gibson, 2007).

Within a long-term project aiming to study the parasite fauna of the marine fishes of the Gulf of Naples through interdisciplinary approaches, unusual philometrid specimens were found infecting the skeletal muscles of the Mediterranean moray. These resulted to be a morphologically different and previously unknown species of the genus *Philometroides* Yamaguti, 1935, herein described based on an integrative taxonomic approach.

## Materials and methods

### Collection data and parasitological study

A total of 32 Mediterranean moray specimens were obtained between July 2021 and March 2023 from professional fishermen operating in 3 different localities of the Gulf of Naples (Nisida Island: 40.7991N, 14.1535E; Pozzuoli: 40.8161N, 14.144E; Procida Island: 40.7494N, 14.0167E) with nets, pots or longlines at depths ranging between 5 and 30 m. Fish were immediately refrigerated and studied within 24 h after death. They were measured (total length – TL) to the nearest 0.1 cm and sex was determined by gonadal examination at necropsy. Then, eyes, skin, gills, mouth cavity, digestive tract (stomach and intestine), liver, heart, gonads, visceral cavity, mesenteries and skeletal muscles of each specimen were examined for parasites under a dissecting microscope (Zeiss Axio Zoom V16, Zeiss, Switzerland) using the methods described in Santoro *et al*. (2022, 2023). When philometrid nematodes were observed in muscles, they were extracted with tweezers, washed in physiological saline solution and preserved in 70% ethanol, 2.5% glutaraldehyde or frozen for subsequent morphological and molecular analyses.

For the light microscopy, nematodes were cleared with Amman's lactophenol, and measurements were obtained using a compound microscope (Axio Imager M1, Zeiss) and a dissecting microscope equipped with the ZEN 3.1 imaging system (Zeiss). Measurements are reported in micrometres unless otherwise indicated and are expressed as mean value ± standard deviation, followed by ranges and the number of measurements for each character in parenthesis. Drawings were made with the aid of a XP PEN Deco 02 drawing tablet and the software Adobe Illustrator and Adobe Photoshop. For the scanning electronic microscopy (SEM), anterior and posterior extremities and intermediate portions of 2 individual nematodes fixed in 2.5% glutaraldehyde were transferred to 40% ethanol (10 min), rinsed in 0.1 M cacodylate buffer, postfixed in 1% OsO4 for 2 h and dehydrated in ethanol series, critical point dried and sputter-coated with platinum. Observations were made using a JEOL JSM 6700F scanning electron microscope operating at 5.0 kV (JEOL, Italy). For the histopathological study, selected samples of skeletal muscles of infected fish were fixed in 10% neutral phosphate-buffered formalin and processed by routine methods into paraffin blocks which were cut into 3 *μ*m-thick sections and stained with haematoxylin and eosin.

### Molecular and phylogenetic analyses

Genomic DNA was extracted from 3 frozen specimens using the Quick-gDNA Miniprep Kit (Zymo Research, USA). The 18S rRNA sequences were amplified and sequenced using the combination of 3 sets of primers [WormA (5′-GCGAATGGCTCATTAAATCAG-3′) and 1270R (5′-CCGTCAATTCCTTTAAGTTT-3′); 1100F (5′-CAGAGATTCGAAGACGATC-3′) and WormB (5′-CTTGTTACGACTTTTACTTCC-3′)] of which 1 was internal pair [930F (5′-GCATGGAATAATGGAATAGG-3′) and 1262R (5′-AACGGCCATGCACCACCACCC-3′)] (Littlewood and Olson, 2001).

Polymerase chain reactions (PCRs) were performed in 25 *μ*L volume containing 1.5 *μ*L of each primer 10 mm, 3 *μ*L of MgCl2 25 mm (Promega, USA), 5 *μ*L of 5× buffer (Promega), 0.6 *μ*L of dNTPs 10 mm (Promega), 0.2 *μ*L of Go-Taq Polymerase (5U *μ*L^−1^) (Promega) and 2 *μ*L of total DNA. PCR cycling parameters for primer sets WormA and 1270R and 1100F and WormB were as follows: 94°C for 2 min, followed by 40 cycles of 94°C for 30 s, 50°C for 30 s, 72°C for 2 min and a final 72°C extension for 7 min. PCR cycling parameters for 930F and 1262R were as follows: 94°C for 5 min, followed by 40 cycles of 94°C for 40 s, 50°C for 40 s, 72°C for 2 min and a final 72°C extension for 10 min.

Successful PCR products were purified using Agencourt AMPure XP (Beckman Coulter, USA), following the standard manufacturer-recommended protocol. Clean PCR products were Sanger sequenced from both strands using an Automated Capillary Electrophoresis Sequencer 3730 DNA Analyzer (Applied Biosystems, USA) and the BigDye® Terminator v. 3.1 Cycle Sequencing Kit (Life Technologies, USA). All 3 sets of primers were used for sequencing to maximize sequences quality. The obtained contiguous sequences were assembled and edited using MEGAX v. 11 (Kumar *et al*., 2018). Sequence identity was verified using the Nucleotide Basic Local Alignment Search Tool (BLASTn) (Morgulis *et al*., 2008).

Sequences representatives of Philometridae were downloaded from GenBank for phylogenetic analysis according to Barton *et al*. (2022), who provided an updated phylogeny of philometrid nematodes based on the 18S rRNA nuclear gene (Table 1). Sequences of analogous length to the ones generated in this study were selected (>1600 bp). Two dracunculoids (family Dracunculoidae Stiles, 1907), namely *Dracunculus medinensis* (Linnaeus, 1758) (MK163617) and *Philonema* sp. (U81574), were selected as outgroups (Sokolov *et al*., 2020; Barton *et al*., 2022). The sequences were aligned using the MAFFT algorithm (Katoh *et al*., 2019) implemented in T-Coffee (Notredame *et al*., 2000), then submitted to the transitive consistency score to verify the reliability of aligned positions and optimize the phylogenetic topology (Chang *et al*., 2015). In total, 55 sequences were analysed. Maximum likelihood (ML) and Bayesian inference (BI) phylogenetic trees were calculated using iQtree v. 1.6.12 (Nguyen *et al*., 2015) and MrBayes v. 3.2.7 (Ronquist and Huelsenbeck, 2003), respectively. The best fitted evolutionary models were HKY + I + G as suggested by JmodelTest v. 2.1.10 (Darriba *et al*., 2012). For the ML analyses, 5000 ultrafast bootstrap approximations were performed to test the phylogenetic reliability. Posterior probability distributions for the Bayesian analysis were generated using Markov Chain Monte Carlo (MCMC) method. MCMC searches were run for 10.000.000 generations on 2 simultaneous runs of 4 chains and sampled every 1.000 generations; the first 25% of samples from the MCMC algorithm were discarded as burn in. The quality of the Bayesian analysis (parameter densities, effective sample size and burn-in) and the chain convergence were examined in Tracer (Rambaut *et al*., 2018). Trees were visualized using Figtree v. 1.4.4 (Rambaut, 2012). The definition of the major clades in all phylogenies of this work follows Barton *et al*. (2022).

**Table 1.** Information about sequences obtained from GenBank used in the phylogenetic analysis

GenBank ID	Parasite species	Host species	Host family (order)	Host habitat	Site of infection	Geographic origin	Reference
JF803946	*Afrophilometra hydrocyoni*	*Hydrocynus forskahlii*	Alestidae (Characiformes)	FW	Fins and nearby muscles	Kenya	Černotíková *et al*. (2011)
DQ442672	*Alinema amazonicum*	*Callophysus macropterus*	Pimelodidae (Siluriformes)	FW	Abdominal cavity; mesentery	Peru	Wijová *et al*. (2006)
JF803939	*Caranginema americanum*	*Caranx hippos*	Carangidae (Carangiformes)	SW	Subcutaneous tissue	USA	Černotíková *et al*. (2011)
DQ442673	*Dentiphilometra lutjani*	*Lutjanus griseus*	Lutjanidae (Perciformes)	SW	Ovary	Mexico	Wijová *et al*. (2006)
MZ274359	*Digitiphilometroides marinus*	*Rachycentron canadum*	Rachycentridae (Carangiformes)	SW	Body cavity	Northern Australia	Barton *et al*. (2022)
MK163617	*Dracunculus medinensis*^a^	*Homo sapiens*	Hominidae (Primates)	T	Subcutaneous tissue	Angola	Unpublished
AB185161	*Margolisianum bulbosum*	*Paralichthys lethostigma*	Paralichthyidae (Pleuronectiformes)	SW	Subcutaneous tissue	USA	Unpublished
DQ442671	*Nilonema senticosum*	*Arapaima gigas*	Arapaimidae (Osteoglossiformes)	FW	Abdominal cavity; swim bladder	Peru	Wijová *et al*. (2006)
MW328559	*Philometra aequispiculata*	*Strongylura marina*	Belonidae (Beloniformes)	SW	Ovary	Florida	Moravec *et al*. (2021)
JF803948	*Philometra bagri*	*Bagrus bajad*	Bagridae (Siluriformes)	FW	Subcutaneous tissue (head)	Kenya	Černotíková *et al*. (2011)
JF803943	*Philometra brevispicula*	*Lutjanus griseus*	Lutjanidae (Perciformes)	SW	Subcutaneous tissue (mouth)	USA	Černotíková *et al*. (2011)
DQ442675	*Philometra cyprinirutili*	*Abramis brama*	Cyprinidae (Cypriniformes)	FW	Abdominal cavity	Czech Republic	Wijová *et al*. (2006)
JF803942	*Philometra diplectri*	*Diplectrum formosum*	Serranidae (Perciformes)	SW	Subcutaneous tissue (head)	USA	Černotíková *et al*. (2011)
JF803928	*Philometra floridensis*	*Sciaenops ocellatus*	Sciaenidae (Acanthuriformes)	SW	Ovary	USA	Černotíková *et al*. (2011)
MZ274354	*Philometra globiceps*	*Uranoscopus scaber*	Uranoscopidae (Trachiniformes)	SW	Ovary	Italy (Ionian Sea)	Barton *et al*. (2022)
MZ274362	*Philometra gracilis*	*Lutjanus johnii*	Lutjanidae (Perciformes)	SW	Tissues behind head	Northern Australia	Barton *et al*. (2022)
JF803916	*Philometra gymnosardae*	*Gymnosarda unicolor*	Scombridae (Scombriformes)	SW	Abdominal cavity	Maldives	Černotíková *et al*. (2011)
MZ274349	*Philometra iraqiensis*	*Liza kluzingeri*	Mugilidae (Mugiliformes)	SW	Ovary	Iraq (Arabian Gulf)	Barton *et al*. (2022)
MH725819	*Philometra kotlani*	*Aspius aspius*	Cyprinidae (Cypriniformes)	FW	Mesentery of ovary	Russia	Sokolov *et al*. (2020)
KP122959	*Philometra lagocephali*	*Lagocephalus lunaris*	Tetraodontidae (Tetraodontiformes)	SW	Abdominal cavity	Southern China	Wang *et al*. (2015)
FJ161972	*Philometra lateolabracis*	*Lateolabrax japonicus*	Lateolabracidae (Perciformes)	SW	Ovary	Japan	Quiazon *et al*. (2008)
JF803945	*Philometra lati*	*Lates niloticus*	Latidae (Perciformes)	FW	Abdominal cavity	Kenya	Černotíková *et al*. (2011)
MZ274356	*Philometra longa*	*Hyporhampus australis*	Hemiramphidae (Beloniformes)	SW	Abdominal cavity	Southern Australia	Barton *et al*. (2022)
FJ161974	*Philometra madai*	*Pagrus major*	Sparidae (Spariformes)	SW	Ovary	Japan	Quiazon *et al*. (2008)
JF803933	*Philometra morii*	*Epinephelus morio*	Serranidae (Perciformes)	SW	Subcutaneous tissue (mouth)	USA	Černotíková *et al*. (2011)
MH930986	*Philometra nattereri*	*Serrasalmus gibbus*	Serrasalmidae (Characiformes)	FW	Stomach wall	Brazil	Negreiros *et al*. (2019)
FJ161975	*Philometra nemipteri*	*Nemipterus virgatus*	Nemipteridae (Perciformes)	SW	Ovary	Japan	Quiazon *et al*. (2008)
MW328560	*Philometra notatae*	*Strongylura notata*	Belonidae (Beloniformes)	SW	Swim bladder	USA	Moravec *et al*. (2021)
AY852267	*Philometra obturans*	*Esox lucius*	Esocidae (Esociformes)	FW	Gill blood vessel	Czech Republic	Wijová *et al*. (2006)
MH725823	*Philometra obturans*	*Esox lucius*	Esocidae (Esociformes)	FW	Gill blood vessel	Russia	Sokolov *et al*. (2020)
JF803929	*Philometra ocularis*	*Epinephelus areolatus*	Serranidae (Perciformes)	SW	Tissues behind eye	New Caledonia	Černotíková *et al*. (2011)
DQ442677	*Philometra ovata*	*Gobio gobio*	Cyprinidae (Cypriniformes)	FW	Abdominal cavity	Czech Republic	Wijová *et al*. (2006)
LC536677	*Philometra pellucida*	*Arothron mappa*	Tetraodontidae (Tetraodontiformes)	SW	Abdominal cavity	Japan	Iwaki *et al*. (2020)
MZ274353	*Philometra rara*	*Hyporthodus haifensis*	Serranidae (Perciformes)	SW	Ovary	Libya	Barton *et al*. (2022)
MH725822	*Philometra rischta*	*Alburnus alburnus*	Cyprinidae (Cypriniformes)	FW	Subcutaneous tissue	Russia	Sokolov *et al*. (2020)
JF803920	*Philometra saltatrix*	*Pomatomus saltatrix*	Pomatomidae (Perciformes)	SW	Ovary	USA	Černotíková *et al*. (2011)
FJ161971	*Philometra sciaenae*	*Nemipterus virgatus*	Nemipteridae (Perciformes)	SW	Ovary	Japan	Quiazon *et al*. (2008)
DQ442674	*Philometra* sp.^b^	*Argyrosomus japonicus*	Sciaenidae (Acanthuriformes)	SW	Abdominal cavity; mesentery	Australia	Wijová *et al*. (2006)
JF803940	*Philometra* sp.	*Mycteroperca microlepis*	Serranidae (Perciformes)	SW	Subcutaneous tissue (mouth)	USA	Černotíková *et al*. (2011)
MZ274351	*Philometra* sp.	*Lutjanus johnii*	Lutjanidae (Perciformes)	SW	Ovary	Northern Australia	Barton *et al*. (2022)
MZ274357	*Philometra* sp.	*Saurida tumbil*	Synodontidae (Aulopiformes)	SW	Ovary	Iraq (Arabian Gulf)	Barton *et al*. (2022)
MZ274363	*Philometra* sp.	*Lutjanus johnii*	Lutjanidae (Perciformes)	SW	Ovary	Northern Australia	Barton *et al*. (2022)
MZ274364	*Philometra* sp.	*Lutjanus johnii*	Lutjanidae (Perciformes)	SW	Ovary	Northern Australia	Barton *et al*. (2022)
JF803944	*Philometra spiriformis*	*Lates niloticus*	Latidae (Perciformes)	FW	Subcutaneous tissue (operculum)	Kenya	Černotíková *et al*. (2011)
JX456388	*Philometra tunisiensis*	*Epinephelus costae*	Serranidae (Perciformes)	SW	Ovary	Turkey	Genc and Keskin (2012)
LC367611	*Philometroides* cf. *branchiostegi*	*Branchiostegus japonicas*	Malacanthidae (Perciformes)	SW	Head tissues	Japan	Abe *et al*. (2019)
PP746031	*Philometroides giginosantoroi* n. sp.	*Muraena helena*	Muraenidae (Anguilliformes)	SW	Skeletal muscles	Italy (Gulf of Naples)	This study
JF803941	*Philometroides grandipapillatus*	*Caranx hippos*	Carangidae (Carangiformes)	SW	Pectoral fin muscle	USA	Černotíková *et al*. (2011)
MH714520	*Philometroides moraveci*	*Perccottus glenii*	Odontobutidae (Gobiiformes)	FW	Subcutaneous tissue (head)	Russia	Unpublished
DQ442676	*Philometroides sanguineus*	*Carassius carassius*	Cyprinidae (Cypriniformes)	FW	Fins and subcutaneous tissue	Czech Republic	Wijová *et al*. (2006)
FJ155811	*Philometroides seriolae*	*Seriola quinqueradiata*	Carangidae (Carangiformes)	SW	Muscles	Japan	Quiazon *et al*. (2008)
MW463876	*Philometroides seriolae*	*Seriola quinqueradiata*	Carangidae (Carangiformes)	SW	NS	Republic of Korea	Unpublished
MZ274350	*Philometroides stomachicus*	*Protonibea diacanthus*	Sciaenidae (Acanthuriformes)	SW	Stomach wall	Northern Australia	Barton *et al*. (2022)
U81574	*Philonema* sp.^a^	NS	NS	NS	NS	NS	Unpublished
JF803923	*Rumai rumai*	*Arapaima gigas*	Arapaimidae (Osteoglossiformes)	FW	Abdominal cavity	Brazil	Černotíková *et al*. (2011)

FW, freshwater; NS, not stated; SW, saltwater; T, terrestrial.

a Outgroup.

b Listed as *Philometra lateolabracis* in Table 1 of Wijová *et al*. (2006).

## Results

### General data

**Figure 1.**
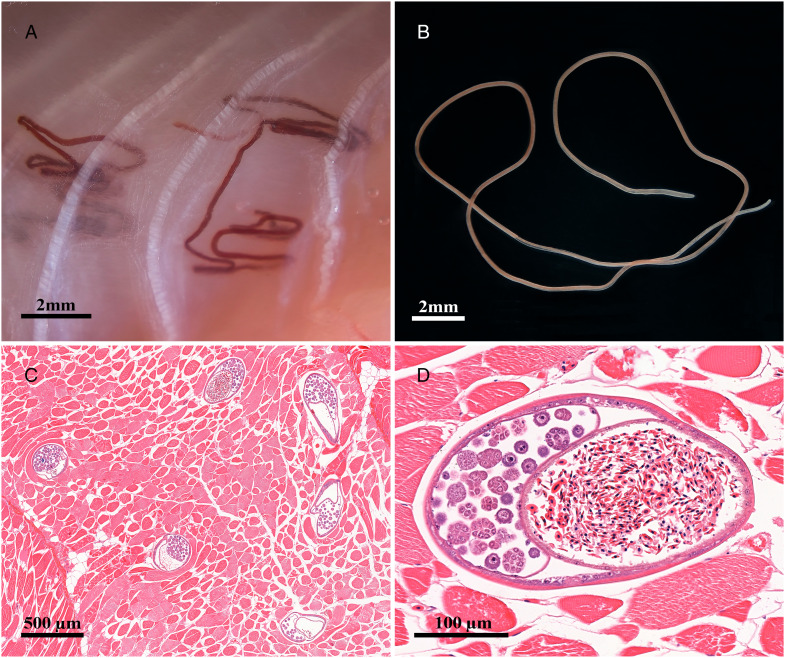
*Philometroides giginosantoroi* n. sp. in the skeletal muscles of the Mediterranean moray. (A) Alive specimens; (B) a 70% ethanol preserved specimen; (C) cross-sections of specimens from the skeletal muscles; (D) magnification of a cross-section of *P. giginosantoroi* n. sp. showing uterus with eggs and intestine filled by host erythrocytes.

A total of 14 philometrid nematodes (all subgravid females) were found in the skeletal muscles (Fig. 1A and B) of 3 out of the 32 (9.4%) Mediterranean morays examined, accounting for 2 females (TL: 70.5 and 54 cm) from Nisida and 1 male (TL: 71 cm) from Pozzuoli. Histological analysis revealed that the parasites elicited no host inflammatory response in the skeletal muscles of its host (Fig. 1C and D). Cross-sections of the parasites showed the presence of red blood cells of the fish host in their intestine, confirming that these philometrids are hematophagous (Fig. 1C and D). Eggs at different development stages were observed in all histological sections inside uteri (Fig. 1C and D).

### Description

Family Philometridae Baylis and Daubney, 1926

Subfamily Philometrinae Baylis and Daubney, 1926

Genus *Philometroides* Yamaguti, 1935

**Figure 2.**
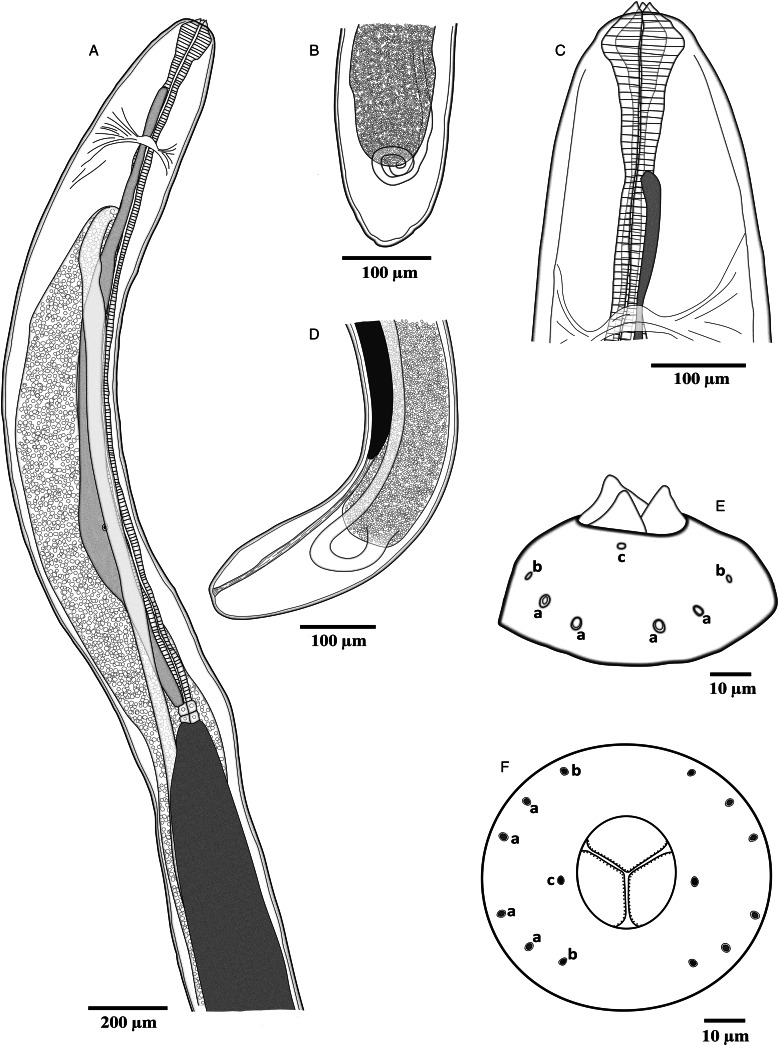
Line drawings of *Philometroides giginosantoroi* n. sp. Anterior (A and C) and posterior (B and D) ends; lateral (E) and apical (F) view of cephalic end. (E, F) (a) Submedian cephalic papilla of external circle; (b) submedian cephalic papilla of internal circle; (c) lateral cephalic papilla of internal circle.

**Figure 3.**
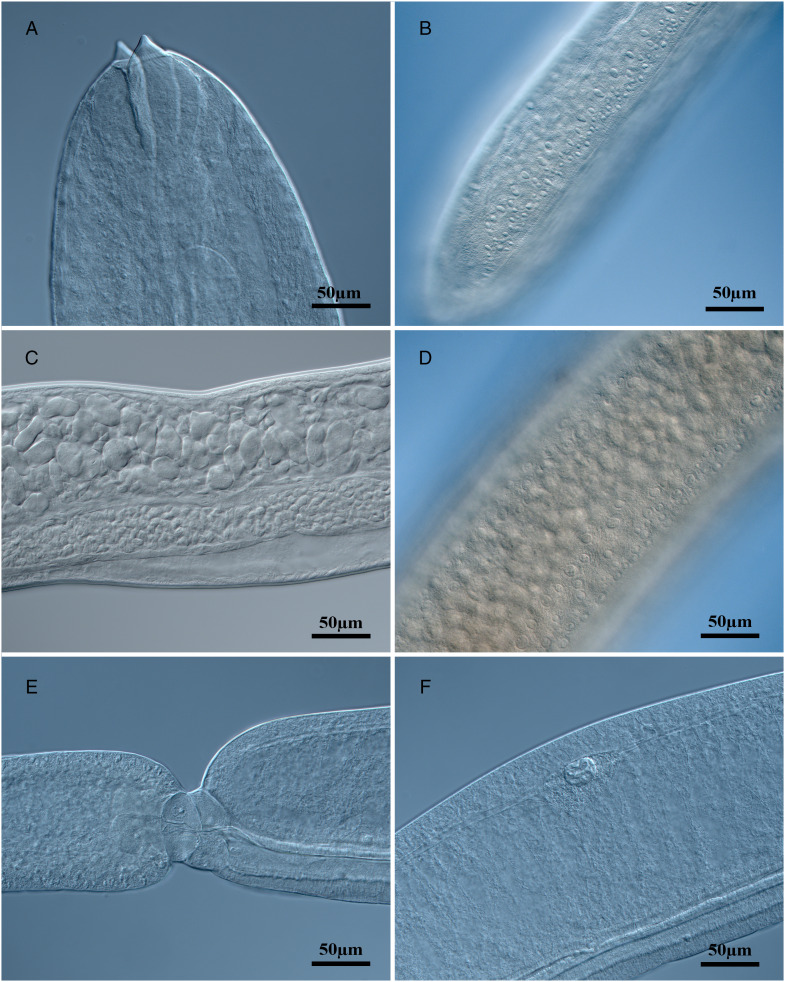
Light micrographs of *Philometroides giginosantoroi* n. sp. (A) Anterior end; (B) posterior end showing cuticular bosses; (C) median portion of the body showing uterus and intestine; (D) median portion of the body showing cuticular bosses; (E) ventriculus of a dissected specimen; (F) oesophageal gland of a dissected specimen showing the cell nucleus.

**Figure 4.**
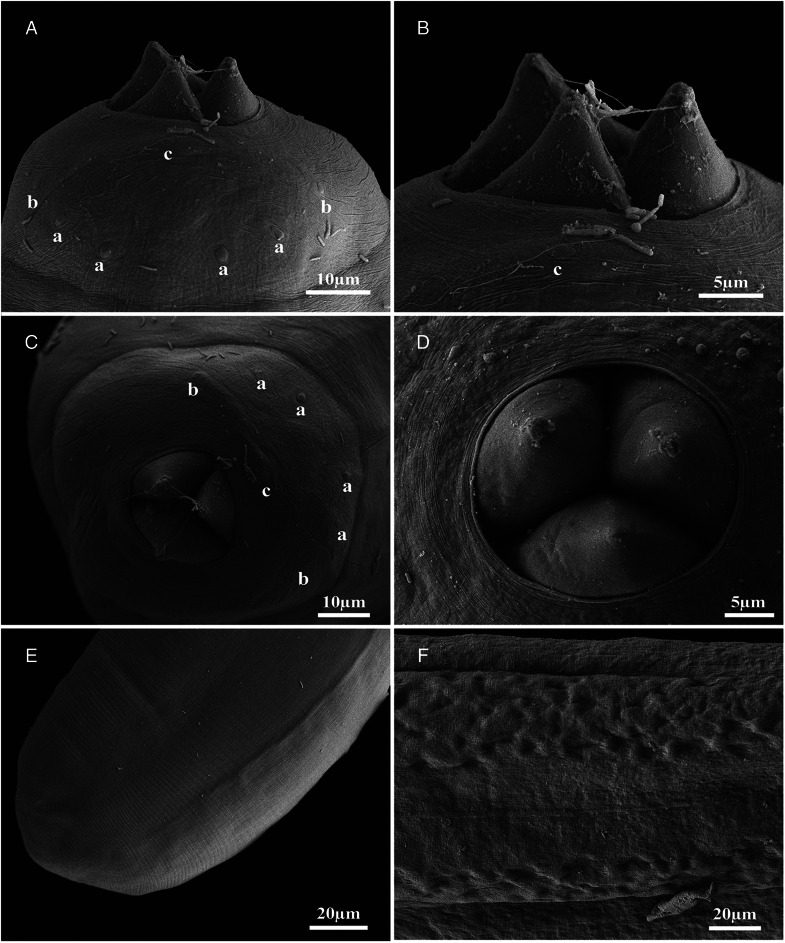
Scanning electron micrographs of *Philometroides giginosantoroi* n. sp. (A) Lateral view of anterior end showing papillae; (B) magnification of lateral view of anterior end showing papilla and oesophageal teeth; (C) apical view of anterior end showing papillae; (D) apical view of oesophageal teeth; (E) posterior end; (F) cuticular bosses in a young individual. (A–C) (a) Submedian cephalic papilla of external circle; (b) submedian cephalic papilla of internal circle; (c) lateral cephalic papilla of internal circle.

*Philometroides giginosantoroi* n. sp. López-Verdejo, Occhibove & Santoro, 2024 (Figs 1–4) in López-Verdejo, Occhibove, degli Uberti, Crocetta & Santoro, 2024

ZooBank LSID: urn:lsid:zoobank.org:act:5190867B-2131-4170-9CED-24017092E460.

Type-host: Mediterranean moray *M. helena* Linnaeus, 1758 (Anguilliformes: Muraenidae).

Type-locality: Off Pozzuoli (40.8161N, 14.144E; Gulf of Naples, Italy, Tyrrhenian Sea, central-western Mediterranean Sea) (collected on July 2021).

Other localities: Off Nisida (40.7991N, 14.1535E; Gulf of Naples) (collected on March 2023).

Site in host: Skeletal muscles.

Prevalence and intensity: Overall prevalence 9.4%; 1–12 (mean 4.6) nematodes per infected fish.

Type-material: Holotype (MHNG-INVE-0159470), 3 paratypes and 2 SEM preparations (MHNG-INVE-0159471) in the Parasite Collection of the Natural History Museum of Geneva in Geneva (Switzerland).

Etymology: The species is named in memory of Luigi (Gigino) Santoro, father of the last author recently passed away.

Description (based on 7 complete and 4 incomplete – with no posterior extremity – ovigerous specimens, and selected portions of 2 incomplete specimens studied by SEM). Body of live specimens reddish, filiform, with rounded anterior and posterior extremities (Fig. 1A and B). Small oval-shaped cuticular bosses 8.2 ± 2.1 (4.5–12.3; *n* = 15) in diameter (Fig. 3B and D), irregularly distributed on ventral surface (observed only in larger specimens) from approximately nerve ring area to posterior extremity, conversely cuticular depressions observed on smaller individuals (Fig. 4F). Body length 62.7 ± 16.9 (42.3–90.4; *n* = 7) mm long, maximum width at middle 208 ± 33.4 (175–250; *n* = 7). Maximum width/body length ratio 1:296 (240–362; *n* = 7). Width of cephalic extremity 116 ± 12.5 (103–141; *n* = 9). Oral aperture circular with 3 sclerotized triangular oesophageal teeth 20 ± 3.5 (15–28; *n* = 21) wide at base, and 14 ± 2.1 (10–18; *n* = 20) height, protruding out of mouth (Figs 2C, 2E, 3A, 4A, 4B, 4C and 4D). Cephalic papillae small, arranged in 2 circles: external circle formed by 4 pairs of submedian papillae; internal circle consisting of 6 papillae (4 submedian and 2 lateral) surrounding oral aperture (Figs 2E, 2F, 4A and 4C). Oesophagus muscular, including anterior inflation 1487 ± 200.1 long (1318–1850), representing 2.4% (2.2–3%) of body length (*n* = 7), anterior oesophageal inflation 79 ± 11.2 (67–95) long, 87 ± 8.8 wide (76–104) (Figs 2C and 3A). Ventriculus opening into intestine through valve, 50 ± 5.9 (40–60; *n* = 9) long, 58 ± 11.4 (44–77; *n* = 9) wide (2A and 2E). Oesophageal gland large, 1341 ± 191.6 (1132–1686; *n* = 9) long, starting short anteriorly nerve ring and extending posteriorly to oesophageal end (Fig. 2A), with a large, cell nucleus 1022 ± 185.4 (890–1303; *n* = 7) from anterior extremity (Fig. 3F). Nerve ring 272 ± 33.4 (234–326; *n* = 9), from anterior extremity. Length of intestinal ligament 323 ± 80.8 (197–422). Vulva and anus absent. Uterus filled with numerous eggs (Figs 2A, 2B, 2D and 3C). Posterior end rounded, 112 ± 21.8 wide (72–142; *n* = 7), without caudal projections (Figs 2B, 2D, 3B and 4E).

Male: not known.

### Molecular and phylogenetic analyses

Three identical 18S sequences (1771 bp) were obtained for *P. giginosantoroi* n. sp. BLASTn queries against the NCBI database showed, among others, a 93.4% identity with *Philometra gracilis* Moravec & Barton, 2016 (|MZ274362; 1818 bp), and 93.2% identity with *Philometra lati* Moravec, Charo-Karisa & Jirků, 2009 (|JF803945; 1739 bp). A representative sequence of *P. giginosantoroi* n. sp. was deposited in GenBank under the accession number PP746031.

**Figure 5.**
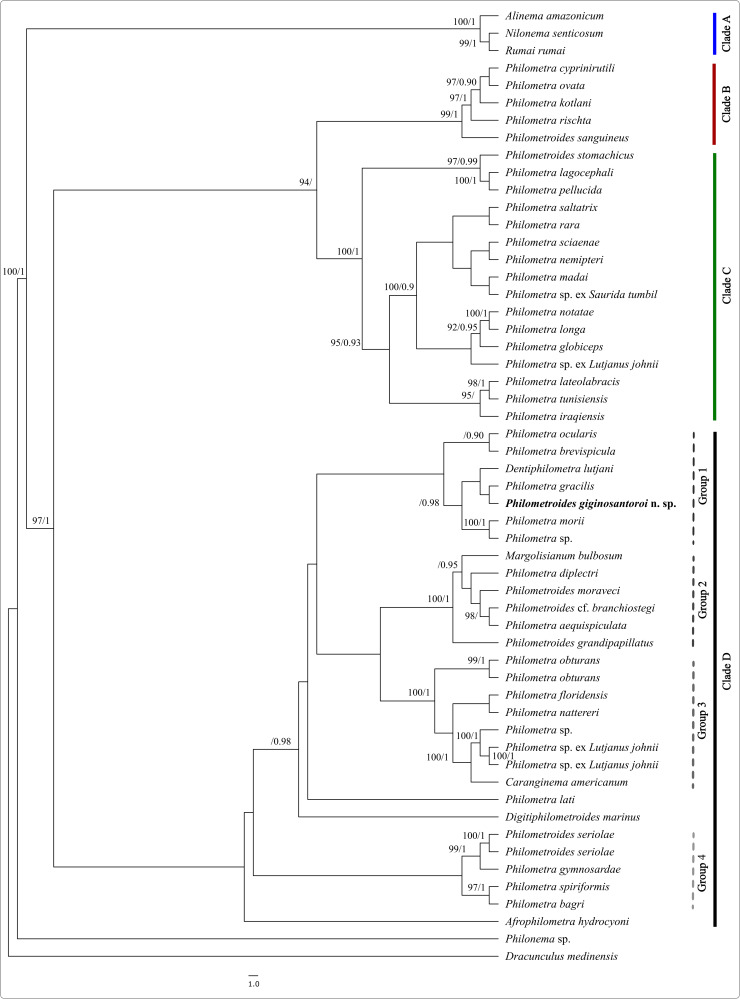
Phylogenetic tree of the family of Philometridae based on partial 18S rRNA sequence alignment of 2079 bp in length. Tree was calculated through maximum likelihood and Bayesian algorithm and shown as Bayesian tree. Ultrafast bootstrap support (maximum likelihood tree) over 90% and posterior probabilities (Bayesian tree) over 0.90 are shown on the nodes (e.g. 90/0.90).

Phylogenetic analyses incorporated 55 sequences ascribed to 53 nominal species (Fig. 5) (alignment length 2079 bp). Topologies of ML and BI trees were identical. Philometridae formed a fully supported monophyletic assemblage. In agreement with previous studies, both ML and BI (Fig. 5) grouped the sequences into 4 fully supported clades (Barton *et al*., 2022; Ailán-Choke *et al*., 2023). Clade A included parasites located either in host body cavity or in subcutaneous tissues of freshwater fishes from South America (Neotropical Region). Clade B represented philometrids of freshwater Cypriniformes from Europe (Palearctic Region), mostly parasitizing host body cavity. Clade C resulted sister to clade B and comprised parasites of a variety of marine fish from different ecoregions; these were mostly found in host ovaries, but also in the body cavity. Clade D, which included the studied *Philometroides* specimens, represented parasites with a diverse array of host taxa, habitat/ecoregion and site of infection of gravid females (Fig. 5). Within clade D, the 4 groups identified in previous phylogenies were also confirmed by our analyses. They accounted for: group (1) parasites of subcutaneous tissues of marine fish; group (2) parasite species belonging to different genera and characterized by a wide range of features; group (3) philometrids with a variety of host orders, site of infections and geographic origins (all *Philometra* spp. except *Caranginema americanum* Moravec, Montoya-Mendoza & Salgado-Maldonado, 2008); group (4) including *Philometra* and *Philometroides* species. Noteworthy, *Afrophilometra hydrocyoni* (Fahmy, Mandour & El-Nafar, 1976) Moravec, Charo-Karisa & Jirků, 2009, *Dentiphilometroides marinus* Moravec & de Buron, 2009 and *P. lati* Moravec, Charo-Karisa & Jirků, 2009 were not assigned to any group within clade D in the previous studies.

*Philometroides giginosantoroi* n. sp. grouped with tissue-infecting philometrids from group 1 of clade D (D1) (Fig. 5). In particular, it seemed closely related to *P. gracilis* Moravec & Barton, 2016 and *Dentiphilometra lutjani* González-Solís, Moravec & Tuz-Paredes, 2007, which were collected from the head tissues and ovary of their marine fish hosts (both Perciformes), respectively.

### Remarks

The genus *Philometroides* includes so far 35 tissue-dwelling species. Out of those, 20 taxa are parasites of freshwater fishes, 12 of marine fishes and 3 of brackish-water fishes (Moravec and de Buron, 2013; Montes *et al*., 2016; Cavalcante *et al*., 2018; Moravec, 2024). *Philometroides giginosantoroi* n. sp. differs from the congeneric species by the presence of sclerotized oesophageal teeth, absent in all species except *Philometroides branchiarum* Moravec & Barton, 2016, a parasite of gill arches of the John's snapper *Lutjanus johnii* (Perciformes: Lutjanidae), described from Australia on the basis of 2 larvigerous females (Moravec and Barton, 2016). However, the females of *P. branchiarum* have a smaller body (6–9 vs 42.3–90.4 mm), a different shape (conical vs triangular shape) and height (3 vs 20.1) of the oesophageal teeth, a smaller maximum width/length ratio of the body (1:28–33 vs 1:240–362), a different morphology and distribution of the cuticular ornamentations, a different distribution of cephalic papillae, a different position of the oesophageal gland (starting just posteriorly to the nerve-ring vs anteriorly to the nerve ring), a longer oesophagus length ratio with respect to the body length (13–17 vs 2.2–3%), a posterior extremity bearing 2 large lateral caudal projections (absent in the newly described species). In addition, the hosts of *P. branchiarum* and of *P. giginosantoroi* n. sp. live in different ecoregions (Indo-Pacific vs Mediterranean) and belong to different orders and families (Perciformes: Lutjanidae vs Anguilliformes: Muraenidae), while the parasite species have different tissue tropisms (arch gills vs skeletal muscles).

Obvious morphological differences also exist between the new species and *Philometroides oveni* Parukhin, 1975, the only congeneric species reported in the Mediterranean Sea (from off Lampedusa). This species infects the oculo-orbit and the eye of *Serranus hepatus* (Perciformes: Serranidae) (Parukhin, 1975; Moravec and de Buron, 2013; Moravec, 2024).

*Philometroides oveni* differs from the new species by having smaller body (28 vs 62.7 mm), absence vs presence of oesophageal teeth, different number and pattern distribution of cephalic papillae (*P. oveni* has 4 pairs of papillae arranged in a single circle) (Parukhin, 1975), maximum width/body length ratio (1:36 vs 1:296), oesophagus/body length ratio (0.03 vs 2.4%), different host orders and families and different tissue tropism.

## Discussion

According to Moravec (2024), the family Philometridae includes 3 subfamilies with 16 genera and 217 valid species: Alineminae (1 genus with 1 species), Neophilometroidinae (1 genus with 2 species) and Philometrinae (14 genera with 214 species). Within Philometrinae, the genera *Philometroides* and *Philometra* are quite similar, with the main features used to distinguish the genera being the presence/absence of cuticular bosses and oesophageal teeth (Moravec, 2006; Anderson *et al*., 2009). The general morphology of *P. giginosantoroi* n. sp. well corresponded to the diagnosis of *Philometroides*, as it showed cuticular bosses and presence of well-developed oesophageal teeth. Therefore, *P. giginosantoroi* n. sp. raises to 218 species of the family and to 36 species of the genus.

Members of Philometridae, moreover, exhibit a high degree of host specificity and tissue tropism (Rasheed, 1963; Ivashkin *et al*., 1971; Moravec, 2004, 2006, 2024; Moravec and Justine, 2009; Moravec and de Buron, 2013; Moravec *et al*., 2019). The discovery of *P. giginosantoroi* n. sp. is also remarkable because it represents the second species of *Philometroides* found in the Mediterranean and in general in European waters and the third species of Philometridae infecting the family Muraenidae worldwide (Moravec, 2024). These are *Philometra gymnothoracis* Moravec & de Buron, 2009, described using 2 gravid females collected from the body cavity of the spotted moray *Gymnothorax moringa* from off the Atlantic coast of South Carolina, USA (Moravec and de Buron, 2009), and *Philometra kidakoi* Moravec, Nagasawa, Nitta & Tawa, 2019, described using a subgravid and an incomplete gravid female from the ovary of the Kidako moray *Gymnothorax kidako* from Western North Pacific Ocean, Japan (Moravec *et al*., 2019). Worth a mention, some fragments of a female of an unidentified species of *Philometroides* were also reported under the skin of a honeycomb moray *Muraena melanotis* off Senegal, although no other information or figures were provided by Campana-Rouget (1956).

Notwithstanding the morphological assignment to the genus *Philometroides*, recent phylogenetic studies suggested that at least some of the morphological characters used to distinguish the genera may be not reliable. This is apparently confirmed also by our phylogenetic analyses, consistent with previous studies and highlighting the presence of 4 main clades (named from A to D), characterized by a combination of features related to the site of infection within the host and the host habitat (freshwater or marine) (Negreiros *et al*., 2019; Barton *et al*., 2022; Ailán-Choke *et al*., 2023). Within clade D, Barton *et al*. (2022) and Ailán-Choke *et al*. (2023) identified 4 sub-clades (named from D1 to D4). The present new species consistently grouped with tissue-infecting philometrids from clade D1, comprising species of 2 additional genera (*Dentiphilometra* and *Philometra*) parasitizing the subcutaneous tissues or muscles of marine fishes. Within this group, *D. lutjani* infects musculature of *Lutjanus griseus* (Lutjanidae), *Philometra ocularis* infects the ocular cavity of Serranidae, *Philometra brevispicula* was found from the mouth and buccal epithelium of *L. griseus*, *P. gracilis* infects the tissues behind the gills of *L. johnii*, *Philometra morii* infects the subcutaneous tissue of buccal cavity and sinuses of *Epinephelus morio* (Serranidae) and *Philometra* sp. infects the subcutaneous tissue of buccal cavity of *Mycteroperca microlepis* (Serranidae). The genus *Dentiphilometra* is mainly characterized by the presence of a sclerotized oral ring armed on its inner surface by numerous small peribuccal teeth in female, while differences between *Philometroides* and *Philometra* were already listed above (Moravec, 2024). Unfortunately, due to the current scarcity of data regarding the host association, life cycle and relevant taxonomic characters of most of philometrids, their phylogenetic relationships is still mostly unresolved (Negreiros *et al*., 2019; Barton *et al*., 2022; Montes *et al*., 2022; Ailán-Choke *et al*., 2023; Moravec, 2024). For instance, not considering the sequence of the present species, the current phylogenetic analysis only included 18S sequences of 6 species of *Philometroides* and other 41 species of philometrids because of the exclusion of shorter sequences, while we included only sequences of comparable length (~1700 bp). Thus, further data on molecular and morphological features are of pivotal relevance to understand the true relationships within the family and shed light on correct genera assignments.

## Data Availability

The authors confirm that the data supporting the findings of this study are available within the article.

## References

[ref1] Abe N, Aono S and Baba T (2019) Site of infection and genetic analysis of *Philometroides branchiostegi*-like nematode found in horsehead tilefish *Branchiostegus japonicus*. Nippon Suisan Gakkaishi 85, 64–66.

[ref2] Ailán-Choke LG, Paschoal F, Couto JV and Pereira FB (2023) On the evolutionary history of Philometridae (Nematoda: Dracunculoidea): integrative taxonomy reveals evidence of character diversification and host–parasite cophylogenetic patterns. Diversity 15, 763.

[ref3] Anderson RC, Chabaud AG and Willmott S (2009) Keys to the Nematode Parasites of Vertebrates, Archival Volume. Wallingford, UK: CAB International.

[ref4] Bartoli P and Gibson DI (2007) The status of *Lecithochirium grandiporum* (Rudolphi, 1819) (Digenea: Hemiuridae), a rarely reported and poorly known species from the Mediterranean moray eel *Muraena helena* L. in the Western Mediterranean. Systematic Parasitology 68, 183–194. 17896187 10.1007/s11230-007-9095-5

[ref5] Bartoli P, Overstreet RM and Gibson DI (2003) First report of a species of *Folliculovarium* Gu and Shen, 1983 (Bucephalidae: Prosorhynchinae) from European marine waters, with the description of *F. mediterraneum* n. sp. Systematic Parasitology 56, 147–154. 14574092 10.1023/a:1026198317540

[ref6] Bartoli P, Gibson DI and Bray RA (2005) Digenean species diversity in teleost fish from a nature reserve off Corsica, France (Western Mediterranean), and a comparison with other Mediterranean regions. Journal of Natural History 39, 47–70.

[ref7] Barton DP, Moravec F, Zhu X and Shamsi S (2022) Phylogenetic relationships of philometrid nematodes (Philometridae Baylis & Daubney, 1926) inferred from 18S rRNA, with molecular characterisation of recently described species. Parasitology Research 121, 127–141. 34825260 10.1007/s00436-021-07373-8

[ref8] Campana-Rouget Y (1956) Parasites de poissons de mer ouest-africains récoltes par J. Cadenat. VI et VII. – Nématodes (2e et 3e notes). VI: parasites de sélaciens iler complément. VII: parasites d'apodes. Bullettin IFAN ser. A 18, 459–466.

[ref9] Cavalcante P, Moravec F and Santos C (2018) The philometrid nematode *Philometroides acreanensis* n. sp. from the stomach wall of the catfish *Pimelodus blochii* in north-western Brazil. Journal of Helminthology 92, 109–115. 28274281 10.1017/S0022149X1700013X

[ref10] Černotíková E, Horák A and Moravec F (2011) Phylogenetic relationships of some spirurine nematodes (Nematoda: Chromadorea: Rhabditida: Spirurina) parasitic in fishes inferred from SSU rRNA gene sequences. Folia Parasitologica 58, 135–148. 21776893

[ref11] Chang JM, Di Tommaso P, Lefort V, Gascuel O and Notredame C (2015) TCS: a web server for multiple sequence alignment evaluation and phylogenetic reconstruction. Nucleic Acids Research 43, W3–W6. 25855806 10.1093/nar/gkv310 PMC4489230

[ref12] Darriba D, Taboada G, Doallo R and Posada D (2012) jModelTest 2: more models, new heuristics and parallel computing. Nature Methods 9, 772. 10.1038/nmeth.2109 PMC4594756 22847109

[ref13] Genc E and Keskin E (2012) *Philometra lateolabracis* (Nematoda: Philometridae) in *Epinephelus costae* (Osteichthyes, Serranidae): first molecular identification of the histozoic parasite from Iskenderun Bay, northeast Mediterranean Sea. AQUA 2012, Abstract No. 695. Prague, Czech Republic: World Aquaculture Society.

[ref14] Ivashkin VM, Sobolev AA and Khromova LA (1971) Camallanata of animals and man and the diseases caused by them. Nauka, Moscow: Osnovy Nematodologii 22.

[ref15] Iwaki T, Tamai K, Ohimoto K, Iwahashi Y, Waki T, Kawano F and Ogawa K (2020) New records of *Philometra pellucida* (Jägerskiöld, 1893) (Nematoda: Philometridae) from the body cavity of *Arothron mappa* (Lesson) and *Arothron nigropunctatus* (Bloch et Schneider) reared in aquariums, with synonymisation of *Philometra robusta* Moravec, Möller et Heeger, 1992. Folia Parasitologica 67, 025. 10.14411/fp.2020.025 33043892

[ref16] Katoh K, Rozewicki J and Yamada KD (2019) MAFFT online service: multiple sequence alignment, interactive sequence choice and visualization. Briefing in Bioinformatics 20, 1160–1166. 28968734 10.1093/bib/bbx108 PMC6781576

[ref17] Kumar S, Stecher G, Li M, Knyaz C and Tamura K (2018) MEGAX: molecular evolutionary genetics analysis across computing platforms. Molecular Biology and Evolution 35, 1547–1549. 29722887 10.1093/molbev/msy096 PMC5967553

[ref18] Littlewood D and Olson P (2001) Small subunit rDNA and the Platyhelminthes: signal, noise, conflict and compromise. In Littlewood D and Bray R (eds), Interrelationships of the Platyhelminthes. London, UK: Taylor & Francis, pp. 262–278.

[ref19] Matić-Skoko S, Tutman P, Bojanić Varezić D, Skaramuca D, Đikić D, Lisičić D and Skaramuca B (2014) Food preferences of the Mediterranean moray eel, *Muraena helena* (Pisces: Muraenidae), in the southern Adriatic Sea. Marine Biology Research 10, 807–815.

[ref20] Montes MM, Plaul SE and Martorelli SR (2016) A new species of philometrid parasite (Nematoda, Philometridae) and histopathological lesions in juvenile croakers, *Micropogonias furnieri* (Desmarest). Journal of Fish Diseases 39, 1053–1059. 26775636 10.1111/jfd.12439

[ref21] Montes MM, Albarracin MA, Barneche J, Croci Y, Balcazar D, Cardarella GFR and Martorelli SR (2022) Molecular phylogenetic relationship between *Philometroides tahieli* (Nematoda, Philometridae) and other philometrids from South America. Parasitology Research 121, 3091–3103. 36125527 10.1007/s00436-022-07652-y

[ref22] Moravec F (2004) Some aspects of the taxonomy and biology of dracunculoid nematodes parasitic in fishes: a review. Folia Parasitologica 51, 1–13. 15139371 10.14411/fp.2004.001

[ref23] Moravec F (2006) Dracunculoid and Anguillicoloid Nematodes Parasitic in Vertebrates. České Budějovice, Czech Republic: Academia.

[ref24] Moravec F (2024) Philometrid Nematodes Parasitic in Fishes. České Budějovice, Czech Republic: Czech Academy of Sciences.

[ref25] Moravec F and Barton DP (2016) New tissue-dwelling species of *Philometra* Costa, 1845 and *Philometroides* Yamaguti, 1935 (Nematoda: Philometridae) from marine perciform fishes off the northern coast of Australia. Systematic Parasitology 93, 623–637. 27522363 10.1007/s11230-016-9657-5

[ref26] Moravec F and de Buron I (2009) Two new species of philometrids (Nematoda: Philometridae) from marine fishes off South Carolina. Journal of Parasitology 95, 722–727. 19115785 10.1645/GE-1866.1

[ref27] Moravec F and de Buron I (2013) A synthesis of our current knowledge of philometrid nematodes, a group of increasingly important fish parasites. Folia Parasitologica 60, 81–101. 23724728 10.14411/fp.2013.010

[ref28] Moravec F and Justine JL (2009) New data on dracunculoid nematodes from fishes off New Caledonia, including four new species of *Philometra* (Philometridae) and *Ichthyofilaria* (Guyanemidae). Folia Parasitologica 56, 129–142. 19606788 10.14411/fp.2009.017

[ref29] Moravec F, Nagasawa K, Nitta M and Tawa A (2019) New records of philometrids (Nematoda: Philometridae) from marine fishes off Japan, including description of *Philometra kidakoi* sp. n. and *Congerinema japonicum* gen. et sp. n. Folia Parasitologica 66, 021. 10.14411/fp.2019.021 31849364

[ref30] Moravec F, Bakenhaster M, Seyoum S and Tringali M (2021) Morphological and genetic description of two new species of philometrid nematodes (Philometridae) parasitic in needlefishes (Belonidae) from estuaries of Florida, USA. Folia Parasitologica 68, 008. 10.14411/fp.2021.008 33871382

[ref31] Morgulis A, Coulouris G, Raytselis Y, Madden TL, Agarwala R and Schaffer AA (2008) Database indexing for production MegaBLAST searches. Bioinformatics 24, 1757–1764. 18567917 10.1093/bioinformatics/btn322 PMC2696921

[ref32] Negreiros L, Tavares-Dias M, Elisei C, Tavares L and Pereira F (2019) First description of the male of *Philometroides acreanensis* and phylogenetic assessment of Philometridae (Nematoda: Dracunculoidea) suggest instability of some taxa. Parasitology International 69, 30–38. 30389617 10.1016/j.parint.2018.10.010

[ref33] Nguyen L-T, Schmidt H, Von Haeseler A and Minh B (2015) IQ-TREE: a fast and effective stochastic algorithm for estimating maximum-likelihood phylogenies. Molecular Biology and Evolution 32, 268–274. 25371430 10.1093/molbev/msu300 PMC4271533

[ref34] Notredame C, Higgins DG and Heringa J (2000) T-Coffee: a novel method for fast and accurate multiple sequence alignment. Journal of Molecular Biology 302, 205–217. 10964570 10.1006/jmbi.2000.4042

[ref35] Ogawa K, Sata N, Sugihara Y, Miyazaki H, Ueno M, Kuramochi S and Shirakash S (2023) Establishment of the life cycle of *Philometroides seriolae* (Nematoda: Philometridae) using surrogate copepod intermediate host. Fish Pathology 58, 15–21.

[ref36] Parukhin AM (1975) *Philometroides oveni* sp. n., a parasite of the sea perch, Paracanthopristis hepatus. Zoologicheskii Zurnal 54, 312–314.

[ref37] Pesando F and Stefanile M (2016) Sperlonga. Le attività di archeologia subacquea dell'Università di Napoli ‘L'Orientale’ nella villa di Tiberio’. Newsletter di Archeologia CISA 7, 205–221.

[ref38] Quiazon K, Yoshinaga T and Ogawa K (2008) Taxonomical study into two new species of *Philometra* (Nematoda: Philometridae) previously identified as *Philometra lateolabracis* (Yamaguti, 1935). Folia Parasitologica 55, 29–41. 18578165 10.14411/fp.2008.005

[ref39] Rambaut A (2012) Figtree v 1.4. 0. 2012 molecular evolution, phylogenetics and epidemiology. Available at http://tree.bio.ed.ac.uk/publications/ (accessed 4 January 2024).

[ref40] Rambaut A, Drummond AJ, Xie D, Baele G and Suchard MA (2018) Posterior summarization in Bayesian phylogenetics using Tracer 1.7. Systematic Biology 67, 901–904. 29718447 10.1093/sysbio/syy032 PMC6101584

[ref41] Rasheed S (1963) A revision of the genus *Philometra* Costa, 1845. Journal of Helminthology 37, 89–130. 13973169 10.1017/s0022149x00019672

[ref42] Ronquist F and Huelsenbeck J (2003) MrBayes 3: Bayesian phylogenetic inference under mixed models. Bioinformatics 19, 1572–1574. 12912839 10.1093/bioinformatics/btg180

[ref43] Sallami B, Ben Salem M, Reynaud C and Capapé C (2014) Diet of Mediterranean moray, *Muraena helena* (Actinopterygii: Anguilliformes: Muraenidae), from the north-eastern Tunisian coast (central Mediterranean). Acta Ichthyologica et Piscatoria 44, 273–283.

[ref44] Santoro M, Palomba M, Aco Alburqueque R and Mattiucci S (2022) Integrative taxonomy reveals *Molicola uncinatus* and *Gymnorhynchus gigas* (Cestoda: Trypanorhyncha) coinfection in the Atlantic pomfret *Brama brama* from the Mediterranean Sea, with notes on the phylogenetic position of *G. gigas* within the family Gymnorhynchidae. Frontiers in Veterinary Science 9, 909163. 35782558 10.3389/fvets.2022.909163 PMC9249017

[ref45] Santoro M, Bellisario B, Fernández-Álvarez FÁ, Crocetta F and Palomba M (2023) Parasites and prey of the nursehound shark *Scyliorhinus stellaris* (Linnaeus, 1758): insights into hidden trophic web interactions in the Mediterranean Sea. Journal of Fish Biology 102, 271–280. 36278782 10.1111/jfb.15259

[ref46] Sokolov S, Kalmykov A and Malysheva S (2020) Phylogeny of dracunculoid nematodes (Chromadorea: Rhabditida: Spirurina: Dracunculoidea) from some Eurasian freshwater fishes. Zootaxa 4858, 521–541. 10.11646/zootaxa.4858.4.3 33056212

[ref47] Wang SX, Li L and Zhang LP (2015) Redescription and genetic characterization of *Philometra lagocephali* Moravec et Justine 2008 (Nematoda: Philometridae) from *Lagocephalus lunaris* (Bloch and Schneider) (Tetraodontiformes: Tetradontidae) in the South China Sea. Acta Parasitologica 60, 395–406. 26204176 10.1515/ap-2015-0055

[ref48] Wijová M, Moravec F, Horák A and Lukeš J (2006) Evolutionary relationships of Spirurina (Nematoda: Chromadorea: Rhabditida) with special emphasis on dracunculoid nematodes inferred from SSU rRNA gene sequences. International Journal for Parasitology 36, 1067–1075. 16753171 10.1016/j.ijpara.2006.04.005

